# Microwave Saturation of Complex EPR Spectra and Free Radicals of Burnt Skin Treated with Apitherapeutic Agent

**DOI:** 10.1155/2013/545201

**Published:** 2013-05-27

**Authors:** Pawel Olczyk, Pawel Ramos, Marcin Bernas, Katarzyna Komosinska-Vassev, Jerzy Stojko, Barbara Pilawa

**Affiliations:** ^1^Department of Community Pharmacy, Medical University of Silesia in Katowice, 41-200 Sosnowiec, Poland; ^2^Department of Biophysics, Medical University of Silesia in Katowice, 41-200 Sosnowiec, Poland; ^3^Institute of Computer Science, University of Silesia, 41-200 Sosnowiec, Poland; ^4^Department of Clinical Chemistry and Laboratory Diagnostics, Medical University of Silesia in Katowice, 41-200 Sosnowiec, Poland; ^5^Center of Experimental Medicine, Medical University of Silesia in Katowice, 40-752 Katowice, Poland

## Abstract

The effect of microwave power on the complex electron paramagnetic resonance spectra of the burn matrix after the therapy with propolis was examined. The spectra were measured with microwaves in the range of 2.2–79 mW. Three groups of free radicals were found in the damaged skin samples. Their spectral lines evolve differently with the microwave power. In order to detect these free radical groups, the lineshape of the spectra was numerically analysed. The spectra were a superposition of three component lines. The best fit was obtained for the deconvolution of the experimental spectra into one Gauss and two Lorentz lines. The microwave power changes also the lineshape of the spectra of thermally injured skin treated with the conventional agent—silver sulphadiazine. The spectral changes were different for propolis and for silver sulphadiazine. The number of individual groups of free radicals in the wound bed after implementation of these two substances is not equal. It may be explained by a higher activity of propolis than of silver sulphadiazine as therapeutic agents.

## 1. Introduction

During high temperature influence on skin, different types of free radicals are formed. The electron paramagnetic resonance (EPR) spectra may be used to solve the problem of the majority of free radical systems in the skin. Each group of free radicals is responsible for the EPR component line in the resultant resonance spectra [1–3]. The information about different free radicals in the thermally damaged skin is fragmentary. The knowledge about the types of free radicals in the skin is important for pharmacotherapy and alternative medicine [2–5]. 

The aim of this work was to check the usefulness of microwave saturation of EPR spectra to obtain information about the number of different groups of free radicals in the matrix of burnt skin during the therapy with propolis. The relative examination of the skin treated with the silver sulphadiazine was performed. The application of microwave effect on the lineshape of the EPR spectra in order to study free radicals in skin samples and free radicals in biological systems was discussed. The proposed innovatory analyses to skin samples are important to check the substances used in alternative medicine.

## 2. Material and Methods

### 2.1. Therapeutic Agents

Propolis ointment was authorized by the National Institute of Hygiene (HZ/06107/00) 1% silver sulfadiazine cream, Lek, Poland. 

### 2.2. Experimental Animals

Ethics Committee of the Medical University of Silesia in Katowice approved the study protocol, based on the application of two 4-month-old, domestic pigs chosen as the experimental animals. The pig body weight at the beginning of the study was approximately 37.5 kg. Mentioned animal species has been proven to be useful for the assessment of wound healing phenomenon because of numerous analogies of human and pig skin, such as the thickness and composition of epidermis; structure and content of keratinous proteins; size orientation and distribution of the vessels; vascularization of the lower region of the follicle; the similar pattern of deep dermal burn healing [6, 7]. The experimental animals were housed according to GLP standards of Polish Veterinary Law. Pigs general condition was estimated by regular control of behavior, body weight, and temperature. Thermal damages were inflicted according to Hoekstra et al. [6] standard model. The animals were divided into control and experimental group, each containing one pig. Control wound was treated with silver sulfadiazine to observe the healing process, occurring after application of the agent of choice in the local burn treatment [8], twice a day, from the 1st to the 5th day of the experiment. Experimental wounds were treated with propolis, twice a day, from the 1st to the 5th day of the experiment. Biopsies were taken five days after burn infliction.

After burns infliction, tissues were rinsed with an antiseptic solution. During the whole experiment, burns were covered with 55–75 mm layer of the propolis and silver sulfadiazine, twice a day. Subsequently, after the treatment with the mentioned therapeutic agents, the injuries were protected with a woven cotton material.

### 2.3. Sample Preparation to EPR Measurements

Tissue material was mechanically purified, premicronized, and weighed. Then tissue samples were homogenized in acetone using a mechanical homogenizer (30000 rpm/min, 4°C, 30 min.) to obtain a homogeneous suspension. Subsequently received tissue homogenates were dried and reweighed. For EPR measurements experimental material was placed in the glass tubes. EPR signals were not detected for the empty glass tubes. The walls of the glass tubes were thin and their external diameter was of 3 mm. The mass of the samples located in the tubes was measured. 

### 2.4. EPR Measurements

#### 2.4.1. Detection of EPR Spectra

An X-band (9.3 GHz) electron paramagnetic resonance spectrometer of Radiopan Firm (Poznan, Poland) was used for detection of free radicals in the skin samples. The EPR spectra were measured with a magnetic modulation of 100 kHz in the wide range of microwave power of 2.2–70 mW. The attenuations were changed from 0 dB to 15 dB. The microwave saturation effect in the EPR spectra, recorded at higher microwave powers, was obtained. 

#### 2.4.2. The Numerical Analysis of the EPR Spectra

The lineshape of the first-derivative EPR spectra of wound bed treated with propolis and silver sulphadiazine was analysed. The experimental EPR spectra were deconvoluted into the component lines. The measured spectra were fitted by a superposition of theoretical lines with Gauss (G) and Lorentz (L) shape. The following sum of theoretical (G, L) lines were tested: GGG, LLL, GGL, and GLL. 

The best fitting of the experimental spectra by the theoretical multicomponent lines was searched. The best result was the fitting with the lowest square error.

The parameters of the individual components in the EPR spectra such as amplitude (*A*), linewidth (Δ*B*_pp_), and *g*-factor were determined. The signal power for the component lines was calculated. The signal power is the percentage fraction of the individual line in the whole spectrum. It was calculated as the percentage fraction of the integral intensity of the individual line in the total integral intensity of the whole spectrum. The signal power gives information about the fraction of the individual type of free radicals in the tested sample. 

The amplitude rises with increasing free radical contents in the sample linewidths depend on magnetic interactions and distances between free radicals. The *g*-factor, in turn, is determined by the type of free radicals [9].

*g*-Values were calculated from the EPR spectrum according to the formula [9, 10]: *g* = *hν*/*μ*_*B*_ *B*_*r*_, where *h* is Planck constant, *ν* is microwave frequency, *μ*_*B*_ is Bohr magneton, and *B*_*r*_ is resonance magnetic field. Microwave frequency (*ν*) was measured by MCM101 recorder of EPRAD Firm (Poznan, Poland). The resonance *B*_*r*_ values were obtained from the EPR lines. 

Free radical concentration was measured by a double integration of the first-derivative EPR spectra and by a comparison to the integral intensity of the reference—ultramarine. The second reference—a ruby crystal—(Al_2_O_3_:Cr^3+^) was also used. 

The numerical analysis was performed for the signal which is given as a time series *f*_*r*_(*x*), *x* = 1 ⋯ *N* represents the function values within the domain (−5, 5) and time step Δ*x* = 10^−3^. The analysed signal is complex and cannot be described by a single basic function. For the analysis, the derivative of basic functions was used. The application is searching for the best match of *f*_*r*_ function using *f*_*p*_ function defined as a composition of basic functions. Before the approximation is performed, the signal is preprocessed by filtering and positioning of the reference point (0, 0). 

For filtering purposes two methods were researched: the moving average filter [11] and the filter based on fast Fourier transformation [12]. The moving average filter removes the noise; however, the signal is deformed, and therefore finding maximal/minimal function values becomes impossible. The *s* parameter defines a number of neighbors taken under consideration while averaging. 

A reference point (0, 0) was found by using a *dx* and *dy* values which define the distance to move the filtered signal *f*_*r*_ according to *X* and *Y* axis. To find the reference values, two heuristic rules were defined: (a) the sum of values of samples separated by *X* axis is close to 0, (b) the absolute value of the sum of functions from the left side of *Y* axis plus the absolute value of the sum of functions from the right side of *Y* should be maximized.

According to the heuristic rule, a simple linear optimization task was defined for *dx* and *dy* values:

(1a) $$\begin{array}{c} \underset{\mathrm{dx},\mathrm{dy}}{\min}\left(\sum_{x=-\infty }^{\infty }f_{r}\left(x+dx\right)+dy\right), \end{array}$$

(1b) $$\begin{array}{c} \underset{\mathrm{dx},\mathrm{dy}}{\max}\left(\left|\sum_{x=-\infty }^{dx}f_{r}\left(x+dx\right)+dy\right|+\left|\sum_{x=dx}^{\infty }f_{r}\left(x+dx\right)+dy\right|\right). \end{array}$$

All undefined values (outside the interval (−5, 5)) equal 0 and do not change the results. Equation (1a) is unaffected by *dx* value and therefore *dy* is estimated using linear approximation. In this case, the simplex method with bounds on the variables was used. Bounds are set empirically for *dx* to (−10, 10) and for *dy* to (−50, 50). Based on estimated *dy* value, the *dx* displacement is estimated according to (1b). The filtered and transformed function *f*_*r*_ is processed by the estimation algorithm.

According to the prepared model of numerical analysis, the signal *f*_*r*_ is approximated by the *f*_*p*_ function. The function *f*_*p*_ is a composition of derivatives of continuous elementary functions. The function *f*_*p*_ was defined as follows:

(2) fp(x)=f1′(x)∘f2′(x)∘⋯∘fi′(x)∘⋯∘fw′(x),fp(x)=p11f1′(x)+p21f2′(x)+⋯+pi1fi′(x)+⋯+pw1fw′(x),

where *p*_*i*1_ is weight of *i*th function, *f*_*i*_′ is derivative of *i*th function, and *w* is quantity of elemental functions, *w* ∈ *N*.

The preliminary analysis of candidate elementary functions *f*_*i*_′ considered sinus, Lorentz, Gaussian, triangle, and quadratic functions. The accuracy was measured using root mean squared error (RMSE) [13]. The error was calculated as follows:

(3) $$\begin{array}{c} \text{RMSE}=\sqrt{\frac{1}{N}\sum_{x=1}^{N}{\left[f_{r}^{}(x)-f_{p}(x)\right]}^{2}}, \end{array}$$

where *N* is the total number of the analyzed values. 

After preliminary research, the Gaussian and Lorentz function was selected as it offers the lowest error rate for the number of function equal or below 3. Therefore, the *f*_*i*_′(*x*) for *i* = 1 ⋯ 3 was defined as

(4) $$\begin{array}{c} f_{i}^{\prime }\left(x\right)\equiv l_{i}^{\prime }\left(x\right)\overset{.}{\vee }f_{i}^{\prime }\left(x\right)\equiv g_{i}^{\prime }\left(x\right),\;\forall i,\\ l_{i}^{\prime }\left(x\right)=\frac{-2\left(x-p_{i2}\right)p_{i3}}{\pi {\left[{\left(x-p_{i2}\right)}^{2}+p_{i3}^{2}\right]}^{2}},\\ g_{i}^{\prime }\left(x\right)=\frac{-x+p_{i2}}{p_{i3}^{3}\sqrt{2\pi }}e^{-{\left(x-p_{i2}\right)}^{2}/2p_{i3}^{2}}. \end{array}$$

The *p*_*ij*_ parameters define the Gaussian and Lorentz function representation. The parameter *p*_*i*2_ defines mean/location parameter while *p*_*i*3_ defines the variation/scale parameter. The optimization task can be defined while based on the defined model. The optimization is based on the number of Lorentz/Gaussian functions. The *p*_*ij*_ parameters were grouped as a *P*_*i*_ matrix:

(5) $$\begin{array}{c} P_{k}=\left[\begin{array}{ccc} p_{11} & p_{21} & p_{31}\\ p_{12} & p_{22} & p_{32}\\ p_{13} & p_{23} & p_{33} \end{array}\right],\;k\in N. \end{array}$$

The best fit was found by minimizing the difference between the real function *f*_*r*_ and *f*_*p*_ function modifying the *P*_*i*_ matrix for given *i* value and Gaussian/Lorentz functions:

(6) $$\begin{array}{c} \underset{k}{\min}\left(\sum_{x=1}^{N}{\left[f_{r}\left(x\right)-f_{p}\left(x,P_{k}\right)\right]}^{2}\right). \end{array}$$

Boundary values were defined as for *p*_*j*1_, *j* = 1 ⋯ *i* parameters; the maximal signal value multiplied by a number of functions was defined for *p*_*j*2_, *j* = 1 ⋯ *i* parameters, which defines the position of function centers and should not exceed the analyzed interval (−5, 5) and *p*_*j*3_, *j* = 1 ⋯ *i* parameters to (0, 30) according to Gaussian and Lorentz functions requirements. To find the best match, the genetic algorithm [14] with Conjugate Gradient Method [15] was implemented. In the first phase, the algorithm finds a rough estimation of the parameters by modifying the values in *P*_*i*_ matrix within given boundaries and then, for best 10 solutions, the Conjugate Gradient Method is executed to minimize the solution error. The population was set to 1000 and *P*_*i*_ matrixes initial values were equally distributed within defined *P*_*i*_ boundaries. Next, 100 epochs are run to find a rough estimation. The best solution from previous generation is moved to the next one while the remaining solutions undergo a mutation and a crossover. The mutation considers changing one of *P*_*i*_ matrix elements by 1/100 of a parameter value range. The crossover is performed by a simple averaging of two samples. The probability of moving to the next epoch is based on the fitness function proposed by Chan and Sudhoff [14].

## 3. Results

The experimental EPR spectra of the skin of burned wounds were best fitted by the superposition of three lines. The shapes of the components were described by Gauss and Lorentz theoretical functions. One Gauss and two Lorentz lines were found in the spectral curves. Different free radical groups are responsible for the individual components in the spectra. The three groups of free radicals with Gauss and two Lorentz lines exist in the tested skin samples. 

**Figure 1:**
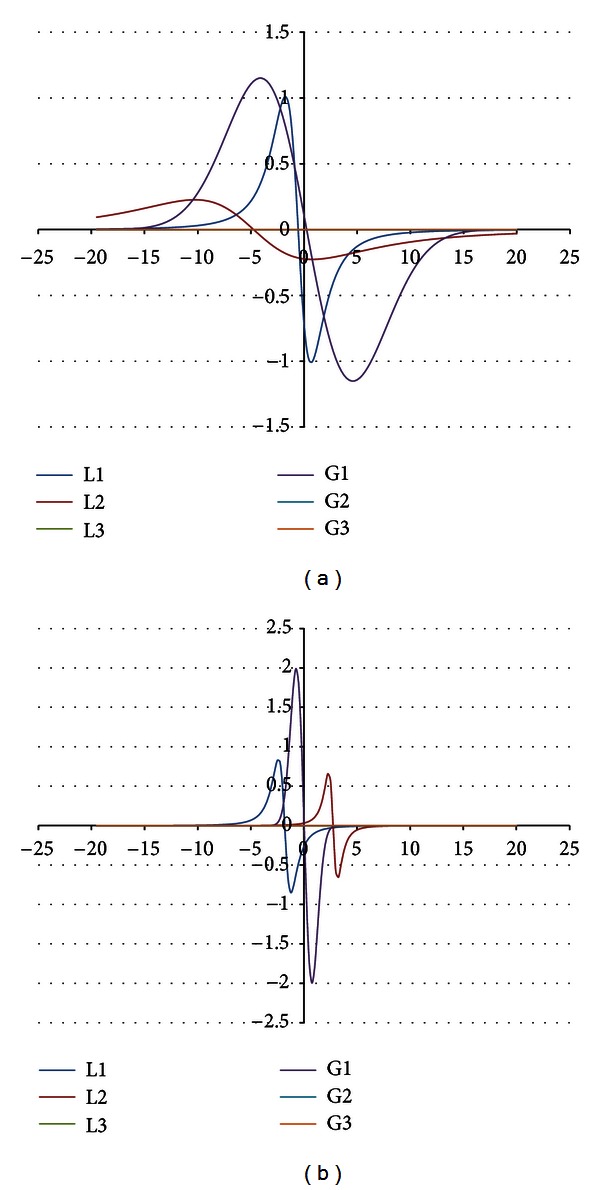
The component EPR lines of the spectrum of thermally damaged skin treated with propolis (a) and silver sulphadiazine (b) for the fitting by summing three Gauss-Lorentz-Lorentz (GLL) lines for the measurements with the microwave power of 2.2 mW (attenuation of 15 dB).

**Figure 2:**
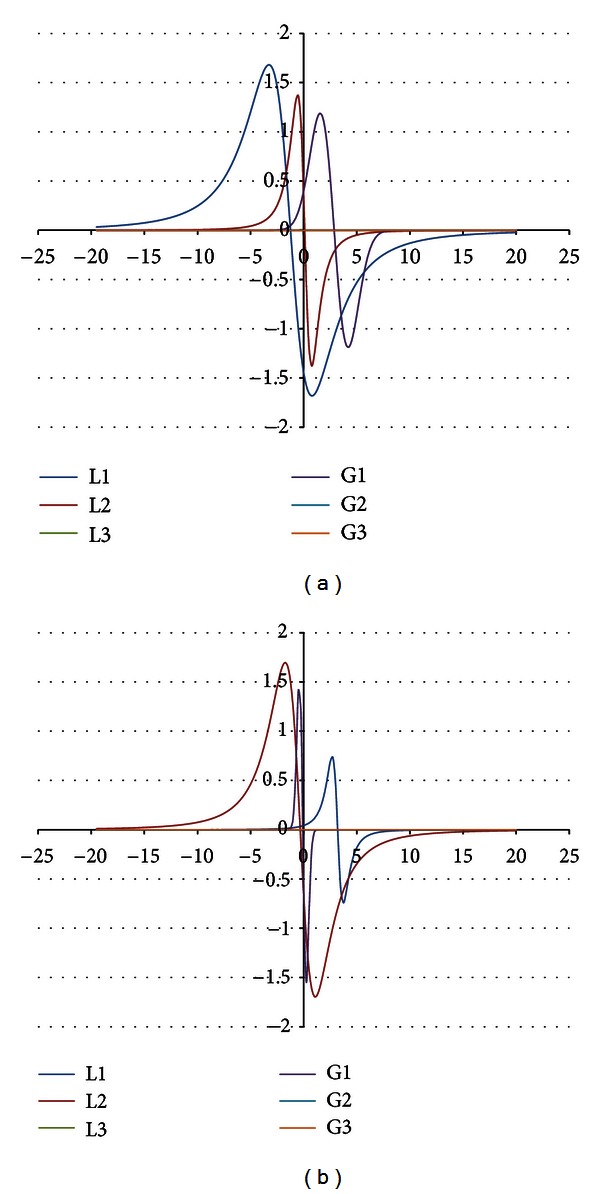
The component EPR lines of the spectrum of thermally damaged skin treated with propolis (a) and silver sulphadiazine (b) for the fitting by summing three Gauss-Lorentz-Lorentz (GLL) lines for the measurements with the microwave power of 7.0 mW (attenuation of 10 dB).

**Figure 3:**
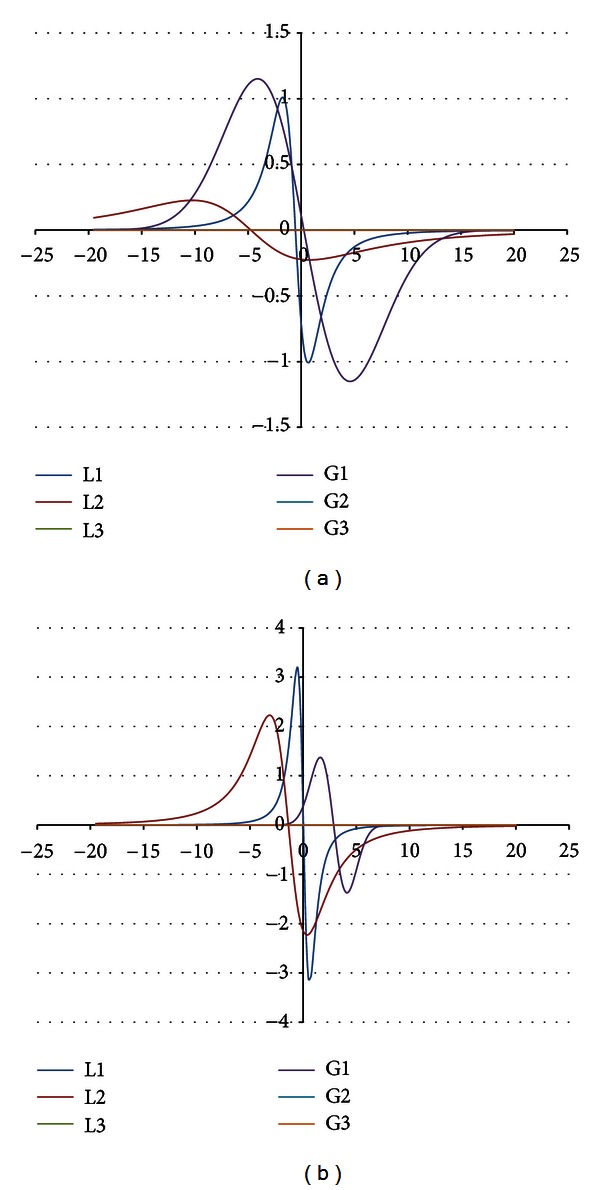
The component EPR lines of the spectrum of thermally damaged skin treated with propolis (a) and silver sulphadiazine (b) for the fitting by summing three Gauss-Lorentz-Lorentz (GLL) lines for the measurements with the microwave power of 11.1 mW (attenuation of 8 dB).

**Figure 4:**
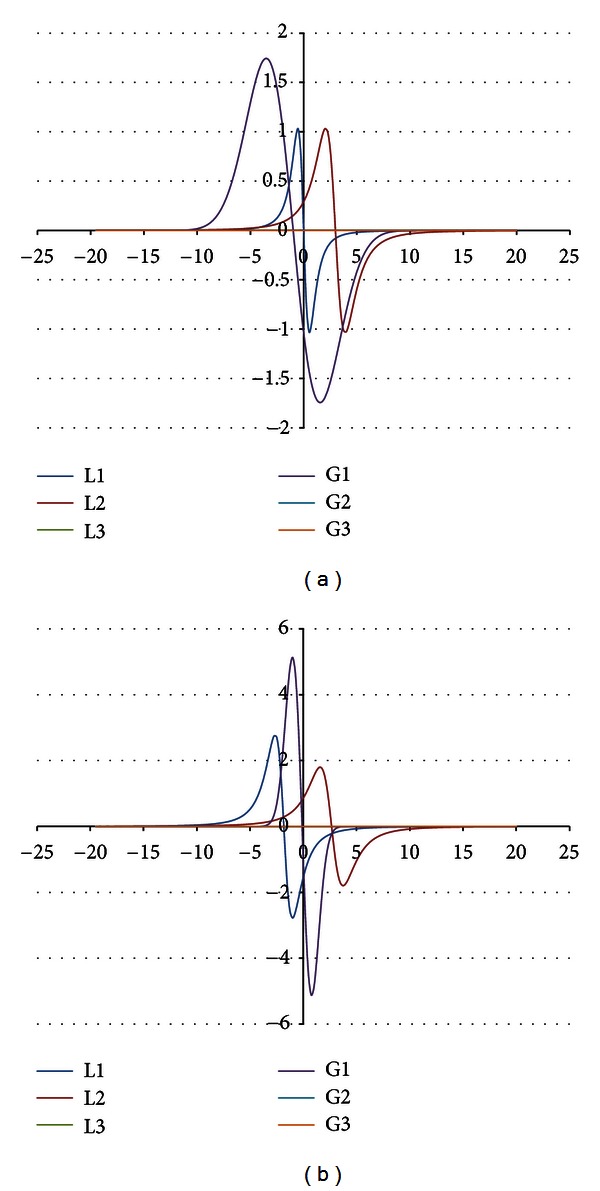
The component EPR lines of the spectrum of thermally damaged skin treated with propolis (a) and silver sulphadiazine (b) for the fitting by summing three Gauss-Lorentz-Lorentz (GLL) lines for the measurements with the microwave power of 22.2 mW (attenuation of 5 dB).

**Figure 5:**
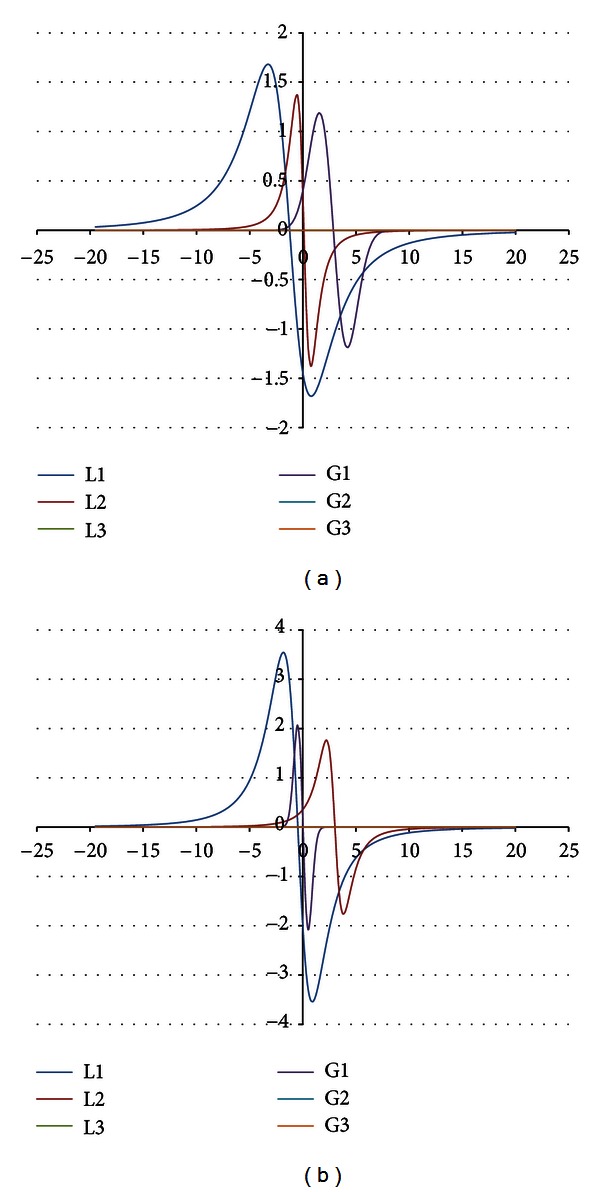
The component EPR lines of the spectrum of thermally damaged skin treated with propolis (a) and silver sulphadiazine (b) for the fitting by summing three Gauss-Lorentz-Lorentz (GLL) lines for the measurements with the microwave power of 35.1 mW (attenuation of 3 dB).

**Figure 6:**
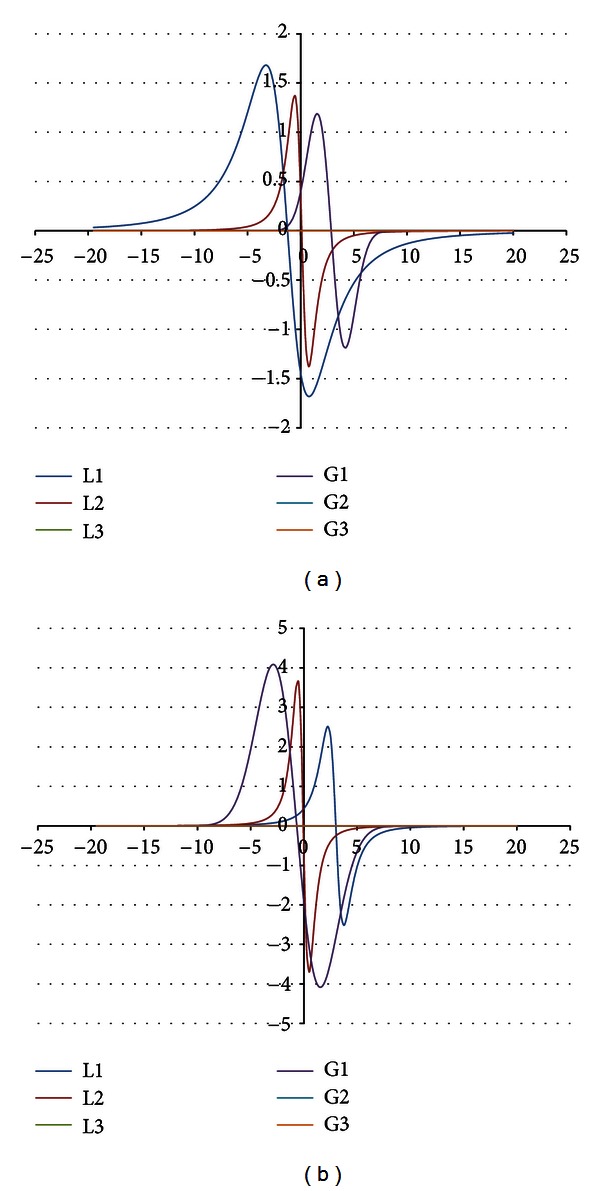
The component EPR lines of the spectrum of thermally damaged skin treated with propolis (a) and silver sulphadiazine (b) for the fitting by summing three Gauss-Lorentz-Lorentz (GLL) lines for the measurements with the microwave power of 55.3 mW (attenuation of 1 dB).

**Figure 7:**
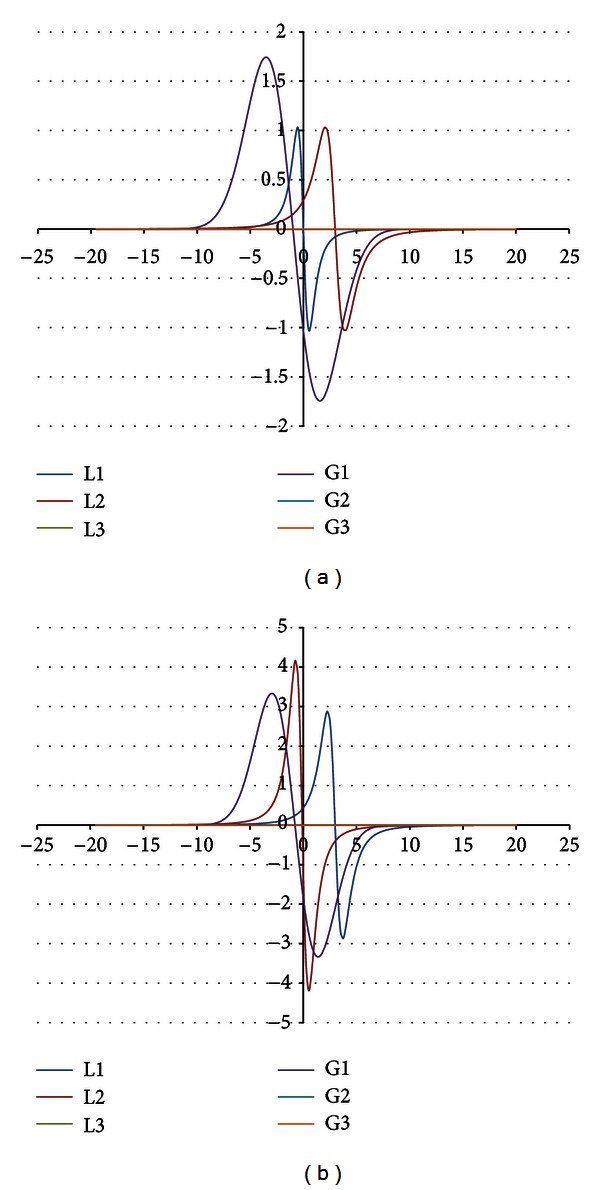
The component EPR lines of the spectrum of thermally damaged skin treated with propolis (a) and silver sulphadiazine (b) for the fitting by summing three Gauss-Lorentz-Lorentz (GLL) lines for the measurements with the microwave power of 70 mW (attenuation of 0 dB).

The components were obtained for the EPR spectra measured with microwave powers of 2.2, 7.0, 11.1, 22.2, 35.1, 55.3, and 70.0 mW. The number of the lines did not depend on the microwave power. The component lines of the EPR spectra of the thermally damaged skin cured with propolis and silver sulphadiazine were presented in Figures 1, 2, 3, 4, 5, 6, and 7. In Figures 1–7(a) the results of the numerical analysis for the skin after the therapy with propolis are shown, while in Figures 1–7(b) the lines for injury therapeutically managed with silver sulphadiazine are presented. The component lines of the spectra measured with different microwave power are presented in these figures. 

A strong influence of the microwave power on the parameters of the component EPR lines was observed for all tested samples. The parameters of the EPR lines: amplitudes (*A*) and linewidths (Δ*B*_pp_), are presented in Table 1 for the injuries treated with propolis and, in Table 2, for the damaged skin treated with silver sulphadiazine. The amplitudes and linewidths change with the microwave power. These changes give way to easier detection of these lines in the resultant spectrum. All measured EPR lines are the broadest ones.

*g*-Values were typical for free radicals paramagnetic centers; they were near 2.00. These values are characteristic for unpaired electrons in organic molecular units.

A different effect of the microwave power on the component lines was observed for the wounded skin samples treated with propolis and silver sulphadiazine (Figures 1–7, Tables 1 and 2). It indicates a different composition of free radical system in these samples.

A lower free radical concentration (13.9 × 10^22^ spin/g) characterizes the burn wound treated with propolis during 5 days rather than the injury treated with silver sulphadiazine (22.99 × 10^22^ spin/g). The free radical concentrations of the individual types of free radicals in the wound therapeutically managed with propolis were also lower than for that treated with silver sulphadiazine.

## 4. Discussion

Different types of free radicals were found in the tested skin samples. Their EPR spectra reveal a multicomponent shape (Figures 1–7). The deconvolution of the EPR spectra into component lines of skin samples is difficult to perform because of high linewidths of the components (Tables 1 and 2). The individual lines overlap in the total spectrum and the resonance magnetic fields are similar to them. As a result, the individual lines are not visible in the EPR spectra and they should be numerically obtained. In this work the numerical procedures of deconvolution of the EPR spectra were additionally made easier by the use of the microwave saturation effect of the EPR lines.

The microwave power increase led to the enhancement of the amplitude of the EPR lines because higher numbers of unpaired electrons come to higher excited energy levels [9]. However, this effect is observed only up to the maximal values of the microwave power and, after higher microwave powers, the saturation of the EPR lines occurs and its amplitude decreases with a former increase of microwave power. The microwave saturation effect—the decrease of amplitudes of the EPR line with increasing microwave power is the result of the inversion of distribution of unpaired electrons on the energy levels [16]. Higher amounts of unpaired electrons are located on higher energy levels rather than on ground levels. The microwave power increases, but the amount of electrons on the ground energy levels is too low. So, the EPR lines are quenched instead of being increased as it is expected. This effect is very useful to find the number of components in the complex EPR spectrum. The described above microwave saturation effect interacts on all types of free radicals in the sample, so it changes their EPR lines as was presented. However, the most important fact is that the microwave saturation effect appears at different microwave powers for the individual component lines. The majority of the microwave saturation effects in different groups of free radicals cause changes of the lineshape of their resultant EPR spectra. The numerical analysis of the EPR spectra, recorded with different microwave powers, and the comparison of their components make the determination of the individual lines easier. The described above information about the microwave saturation effect on EPR spectra was used in this work to analyse different groups of free radicals in burn wound after treatment with propolis. Our expectation is confirmed the three groups of free radicals and their three EPR lines were obtained by mathematical procedures. The number of the EPR lines is stable independently on the microwave power. (Figures 1–7). As it was expected, the parameters of the lines strongly changed with the microwave power (Tables 1 and 2). The fractions of the individual free radical lines in the spectra strongly evolved. The Gauss lines may be used to detect localized stable free radicals while the Lorentz lines give information mainly about delocalized unpaired electrons. The microwave saturation effect was helpful in both kinds of tested samples, the injured skin treated with propolis and silver sulphadiazine. The electron paramagnetic resonance spectroscopic method, the microwave saturation effect, and the numerical analysis of the lineshape of the EPR spectra are proposed not only for the test of propolis influence on the skin but also they may be applied to examine free radicals in the drugs or tissues with several types of free radicals. The performed studies broaden our knowledge about the influence of propolis on free radicals in skin but the practical meaning of this study is equally important. The methods of the free radical examinations to alternative medicine purposes were presented. 

## 5. Conclusions

The advanced spectroscopic method was applied to examine different groups of free radicals in thermally damaged skin treated with propolis. It was proved that the numerical analysis of the lineshape, continuously saturated by microwaves EPR spectra, is useful to determine the number of groups of free radicals in applied samples. These studies broaden our earlier numerical analysis of the EPR spectra measured only with one low microwave power. The microwave saturation of the complex EPR spectra is helpful to examine the multicomponent structure of free radicals in skin samples because of a different effect of the microwave power on the individual component spectral lines. The analysis for propolis was compared with the results of studies for the multicomponent spectra of the standard substance such as silver sulphadiazine. The EPR spectra were best fitted by a superposition of one Gauss and two Lorentz lines. The numerical analysis and microwave saturation of EPR spectra of burnt skin pointed out that the same number of groups of free radicals exists in the skin treated with propolis and silver sulphadiazine. However, the amount of free radicals of different groups in these samples is different. It is expected that it is the reason of a better therapeutic impact of propolis (containing compounds responsible for antioxidant, antimicrobial, anti-inflammatory, antigenotoxic, antiangiogenic, and anticancer properties [17]) on the injured skin than of silver sulphadiazine, which was described in the other work [18]. These studies confirmed the usefulness of both the numerical analysis of the lineshape of EPR spectra and the microwave saturation of the spectra in the examination of complex system of free radicals in skin. The application of the tested methods may be broadened to other biological samples important in alternative medicine. 

## Figures and Tables

**Table 1:** The parameters of the component lines of the EPR of thermally damaged skin treated with propolis fitted by three GLL lines.

*M* [mW]	Parameters	G1	L1	L2	S
2.2	*A* [a.u.]	2.30	2.02	0.45	674.6
	Δ*B*_pp_ [mT]	8.5	2.5	11	
	Signal power	58.4	22.8	18.8	

7.0	*A* [a.u.]	2.37	3.36	2.74	498.1
	Δ*B*_pp_ [mT]	2.75	4	1.25	
	Signal power	18.7	63.7	17.6	

11.1	*A* [a.u.]	2.35	2.10	0.55	237.5
	Δ*B*_pp_ [mT]	8.7	2.5	13	
	Signal power	56.4	24.5	19.1	

22.2	*A* [a.u.]	3.49	2.05	2.06	269.6
	Δ*B*_pp_ [mT]	5	1	2	
	Signal power	64.8	12.9	22.3	

35.1	*A* [a.u.]	2.40	3.35	2.70	542.9
	Δ*B*_pp_ [mT]	2.70	4.2	1.20	
	Signal power	18.5	64.9	16.6	

55.3	*A* [a.u.]	2.45	3.38	2.80	1013.1
	Δ*B*_pp_ [mT]	2.80	3.8	1.50	
	Signal power	16.5	63.6	19.9	

70.0	*A* [a.u.]	3.52	2.01	2.12	11831.5
	Δ*B*_pp_ [mT]	5.3	0.9	2.2	
	Signal power	62.5	14.0	23.5	

G, L: Gauss and Lorentz lines, respectively. S: standard deviation for the fitting. *M*: the microwave power.

**Table 2:** The parameters of the component lines of the EPR spectra of thermally damaged skin treated with silver sulphadiazine fitted by three GLL lines.

*M* [mW]	Parameters	G	L1	L2	S
2.2	*A* [a.u.]	3.99	1.67	1.31	101.9
	Δ*B*_pp_ [mT]	1.5	1.25	1	
	Signal power	54.2	29.4	16.4	

7.0	*A* [a.u.]	2.97	1.48	3.39	136.0
	Δ*B*_pp_ [mT]	0.75	1	2.75	
	Signal power	10.1	12.5	77.4	

11.1	*A* [a.u.]	2.73	6.31	4.45	132.6
	Δ*B*_pp_ [mT]	2.5	1	3.5	
	Signal power	15.9	27.1	57.0	

22.2	*A* [a.u.]	10.26	5.52	3.59	253.2
	Δ*B*_pp_ [mT]	1.75	1.75	2.25	
	Signal power	40.8	31.9	27.3	

35.1	*A* [a.u.]	4.15	7.07	3.51	219.0
	Δ*B*_pp_ [mT]	1	2.75	1.5	
	Signal power	9.3	70.1	20.6	

55.3	*A* [a.u.]	8.15	5.03	7.34	221.5
	Δ*B*_pp_ [mT]	4.5	1.5	1	
	Signal power	59.6	19.7	20.7	

70.0	*A* [a.u.]	6.66	5.75	8.35	226.4
	Δ*B*_pp_ [mT]	4.5	1.5	1.25	
	Signal power	49.6	22.2	28.2	

G, L: Gauss and Lorentz lines, respectively. S: standard deviation for the fitting. *M*: the microwave power.
